# Occlusal and Cephalometric Outcomes of Cleft Orthognathic Surgery: A Retrospective Cohort Study

**DOI:** 10.1002/cre2.70019

**Published:** 2024-11-04

**Authors:** Daniel Stonehouse‐Smith, Aida N.A. Abd Rahman, Victoria Beale, Haydn Bellardie

**Affiliations:** ^1^ Centre for Craniofacial & Regenerative Biology King's College London London UK; ^2^ Faculty of Dentistry Universiti Teknologi MARA Sungai Buloh Selangor Malaysia; ^3^ North West England, The Isle of Man and North Wales Cleft Network Royal Manchester Children's Hospital Manchester UK; ^4^ Faculty of Dentistry University of the Western Cape Cape Town South Africa

**Keywords:** cleft, occlusion, orthodontics, orthognathic surgery

## Abstract

**Objectives:**

Aberrant facial growth in individuals affected by orofacial clefts can result in maxillary retrusion and class III malocclusion, with a proportion requiring surgical correction at cessation of growth. This study aimed to evaluate occlusal and cephalometric outcomes of combined orthodontic‐orthognathic treatment.

**Material and Methods:**

Retrospective cohort study in a United Kingdom cleft center. Participants included twenty‐seven patients (20 males, 7 females) with cleft (n = 16 UCLP :7 BCLP :4 ICP) who consecutively underwent combined surgical treatment for Class III malocclusion between January 2013 and December 2017. Records were collected pre‐treatment (T0), pre‐surgery (T1) and at debond (T2). Models were scored using the Peer Assessment Rating (PAR) index and cephalometric radiographs were traced and analyzed. Outcomes were assessed by an independent rater.

**Results:**

Mean age at surgery was 21.4 years (SD 4.9). Le Fort 1 advancement (mean 6.1 mm, SD 4.0) was performed in all cases. Additional procedures included mandibular setback (n = 2), cortico‐cancellous bone grafting (n = 6) and genioplasty (n = 1). Mean T0 PAR score was 44.8 (SD 11.7), reducing to 3.6 (SD 2.0) at T2, indicating a mean % PAR score reduction of 91.6% (SD 4.7). Class III skeletal profiles improved from a mean T0 ANB of −2.1º (SD 2.2), to 2.8º (SD 1.6) at T2. Mean T0 overjet was −3.3 mm (SD 2.3), increasing to 2.6 mm (SD 1.3) at T2.

**Conclusion:**

Cleft orthognathic surgery differs in complexity and approach to routine orthognathics, however, these results demonstrate that occlusal outcomes can still be comparable with non‐cleft populations. Outcome data can be used for comparison with other centers providing cleft orthognathic treatment.

## 1 Introduction

Individuals born with cleft lip and/or palate (CL/P) undergo numerous surgical procedures in early infancy and throughout childhood to address functional, psychosocial, and esthetic concerns (Dudding, Martin, and Popat 2023). This may culminate in the need for orthognathic surgery at the cessation of growth to correct an underlying imbalance in the skeletal relationship (Posnick and Ricalde 2004). The craniofacial morphology of this population is considered to differ significantly from those without a cleft, often presenting with maxillary retrusion, which can be complicated by bimaxillary retroclination and increased chin protrusion (Küseler et al. 2021). Maxillary hypoplasia as a clinical feature has been attributed to the restriction of maxillary growth caused by scarring from primary surgical cleft repair (Ross 1987; Mars et al. 1987).

Orthognathic surgery will be indicated for a proportion of patients with CL/P who present with a significant class III skeletal relationship at the cessation of growth. This population has been estimated at between 10% (Ross 1987; DeLuke et al. 1997; Friede, Lilja, and Lohmander 2012) and over 75% of patients with a cleft, depending upon type, with the proportion of patients with bilateral clefting being at the higher end of this estimate (Daskalogiannakis and Mehta 2009; Good, Mulliken, and Padwa 2007). These figures also vary widely by the center involved (Mølsted et al. 2005) and may be attributed to differing surgical protocols and criteria for undergoing osteotomy. Where possible, accommodating the underlying skeletal base may be appropriate for individuals with acceptable facial esthetics and milder class III skeletal malocclusion. Some centers report favorable but varying responses to maxillary protraction in adolescence which may reduce the need for orthognathic surgery (Susami et al. 2014). Distraction osteogenesis has also been advocated as an alternative to Le Fort 1 advancement in treating maxillary deficiency (Jamilian et al. 2018). Patients with a more severe class III discrepancy may be beyond the scope of orthodontic camouflage. The decision to carry out orthognathic surgical correction should therefore follow from a multidisciplinary assessment (Dudding, Martin, and Popat 2023).

The Peer Assessment Rating (PAR) Index can be used as a standardized measure of occlusal anomalies, with pre‐ and post‐treatment scores reflecting the improvement and success of treatment (Richmond, Shaw, O'Brien, et al. 1992). Great improvement is considered to be a PAR score reduction ≥ 22 while improvement (< 22 and ≥ 30%) and worse/no improvement (< 22 and ≤ 30%) can also be categorized (Richmond, Shaw, Roberts, et al. 1992). There are no recognized standards for PAR score reduction in patients undergoing cleft orthognathic surgery; however, a comparable United Kingdom cleft center reported a 71% PAR score reduction for a cohort of 21 patients undergoing combined orthognathic treatment (Rolland and Deacon 2014). Previously published outcomes for patients with unilateral cleft lip and palate (UCLP) treated with orthodontics alone reported mean PAR score reductions of 69% (Deacon et al. 2007) and 84.3%, respectively (Stonehouse‐Smith et al. 2022). United Kingdom national standards in non‐cleft patients suggest that 75% of cases should exhibit a reduction in PAR score greater than 70%, with 3% being worse or no different (McMullan et al. 2003). Currently, the PAR index is still used as the only valid measure of cleft‐related orthodontic treatment outcome (Jones et al. 2014). Therefore, the purpose of this study was to evaluate the occlusal and cephalometric outcomes for a cohort of patients undergoing cleft orthognathic surgery in a single United Kingdom cleft center.

## 2 Methods

### 2.1 Design

This was a retrospective cohort study.

### 2.2 Population

A convenience sample comprising patients with any form of CL/P consecutively treated with fixed appliances and undergoing orthognathic surgery between January 1, 2013 and December 31, 2017. The sample included patients of any ethnic background who had completed the cleft orthognathic pathway in this period. All patients were eligible for orthognathic surgery on the basis of the Index of Orthognathic Functional Treatment Need (IOFTN) score 5.1 for defects of cleft lip and palate (Ireland et al. 2014). Standard exclusion criteria (Allori et al. 2017) were applied for those given a syndromic diagnosis, atypical clefts, and those with late entry or transfer of care to the service.

### 2.3 Setting

All patients were treated at a regional tertiary care cleft center in the United Kingdom. All care was provided by the same cleft surgeon and orthodontist and funded by the United Kingdom National Health Service.

### 2.4 Intervention

The standard treatment protocol included a phase of presurgical orthodontic treatment with pre‐adjusted edgewise labial fixed appliances of MBT prescription with 0.022 × 0.025‐inch bracket slots. The surgery involved Le Fort I maxillary advancement osteotomy with rigid internal fixation. An iliac crest origin cortico‐cancellous bone graft was placed at the osteotomy site in cases where the surgeon felt that bone contact in the final position was suboptimal. Rotation of the maxilla was also undertaken for all patients to achieve posterior impaction. Bimaxillary osteotomy was undertaken where there was a significant mandibular contribution to the malocclusion, such as asymmetry. Postsurgical orthodontic treatment was then completed to enable finishing and detailing of the occlusion. Standard retention protocol included vacuum‐formed polypropylene retainers and a lower canine‐to‐canine bonded retainer. An upper bonded retainer was used if significant rotations were corrected in the upper labial segment.

### 2.5 Outcome Measures

Orthodontic study models were scored with the PAR index (Richmond, Shaw, O'Brien, et al. 1992) as the primary outcome measure. Study models and lateral cephalogram radiographs were taken before the start of orthodontic treatment (T0), immediately presurgery (T1), and postsurgery at the time of orthodontic debond (T2). All radiographs were taken using the same cephalostat and standardized exposure settings. Study models were scored at both T0 and T2 by an external assessor (A.A) independent of the treatment process who had undergone training and calibration in the use of the PAR index. Secondary outcomes included both hard and soft tissue cephalometrics. Cephalometric radiographs were digitally traced at all three timepoints by the outcome assessor and analyzed using Dolphin imaging software (Patterson Dental Supply, Inc.). Anteroposterior position of the maxillary incisors was quantified by measuring the amount of overjet (OJ) from the most proclined maxillary incisor tooth. Data on the total number of orthodontic appointments and duration of treatment in fixed appliances were also extracted from clinical records, with treatment time calculated from the date of presurgical orthodontic bond up until removal of the fixed appliances following surgery.

### 2.6 Data Management

Data collection was completed using a pre‐piloted Microsoft Excel (Microsoft, Redmond, WA) spreadsheet. All patient data were recorded anonymously by allocating a unique identifier, with data presented in aggregate. Data analyses were carried out by authors independent of the treatment process (A.N.A.A.R. and D.S.S.).

### 2.7 Ethical Approval

This project was primarily undertaken as part of service evaluation and quality improvement. All records had been taken as part of routine clinical practice and were not for the purposes of research. Ethical approval was therefore not sought but registration and oversight were provided by the local clinical audit team. The principles of the Declaration of Helsinki were observed. Explicit written consent was obtained for the publication of patient images.

### 2.8 Statistical Analyses

Descriptive statistics and summary values were calculated. The repeatability of PAR scoring was determined by random rescoring of 30 sets of study models with a 1‐month interval as part of a larger cohort study (Stonehouse‐Smith et al. 2022). Intra‐rater reliability was measured using intraclass correlation coefficient (ICC) with a two‐way mixed‐effect, single‐measure, absolute agreement model. One‐way ANOVA tests were used to assess the effect of primary cleft diagnosis and type of surgical intervention on the final PAR score (T2) and PAR score reduction (%). A *p* value of 0.05 was considered statistically significant. Statistical analyses were performed using SPSS software version 29.0.2.0 (IBM, SPSS Inc., USA).

## 3 Results

### 3.1 Baseline Characteristics

The sample comprised 27 consecutively treated patients with no exclusions made. Of this sample, 20 (74%) were male and seven (26%) were female. There were 16 (59%) diagnoses of unilateral cleft lip and palate (UCLP), seven (26%) bilateral cleft lip and palate (BCLP), and four (15%) isolated cleft palate (ICP) who underwent combined orthodontic‐orthognathic treatment. All patients had a Le Fort 1 osteotomy with maxillary advancement. The mean amount of advancement was 6.1 mm (SD 4.0). Two patients underwent this in combination with mandibular setback osteotomy (7%), with one also having a genioplasty at the time of the bimaxillary procedure. There were six (22%) patients who required cortico‐cancellous bone grafting. The mean age at the time of surgery was 21.4 years (SD 4.9, range 16.3–39.4). The mean duration of treatment of combined orthodontic‐orthognathic treatment was 20.4 months (range 7–54) over a mean of 21.8 appointments (range 12–45). It is noted that some patients only required simple alignment before orthognathic surgery and so the presurgical orthodontic phase (T0–T1) may only have been several appointments in duration.

### 3.2 Reliability

Intra‐rater reliability of PAR scoring was good at ICC 0.840, 95% CI [0.231, 0.981]. *F* Test with true value 0 was statistically significant, *F*_1_,_4_ = 13.069, *p* = 0.014. Cronbach's *α* = 0.923 (Stonehouse‐Smith et al. 2022).

### 3.3 Occlusal Outcomes

**Figure 1:**
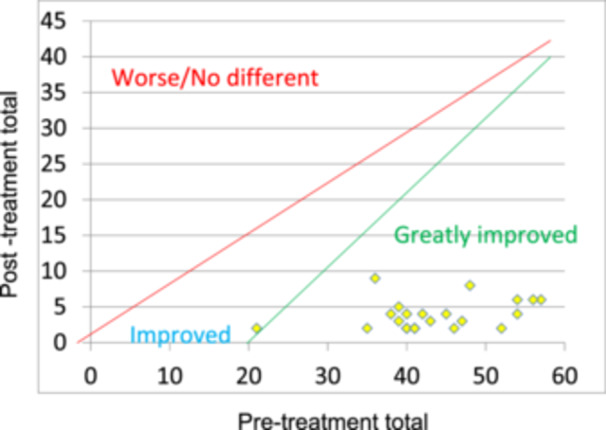
Nomogram for assessment of improvement in PAR score. Please note some patients had identical PAR scoring at T0 and T2 so some points are superimposed.

The mean starting PAR score for all patients was 44.8 (SD 11.7) as detailed in Table 1. Following surgery, this reduced by a mean of 41.2 (SD 11.7) to 3.6 (SD 2.0) which equates to a mean percentage reduction in PAR score of 91.6% (SD 4.7). There were 26 patients (96%) who showed “great improvement” and one patient who showed “improvement” as their starting PAR score was < 22. This is demonstrated in the nomogram in Figure 1. The median PAR efficiency factor (reduction in PAR score divided by treatment time in months) was 1.79. When comparing by cleft diagnosis, the mean final PAR score (T2) in cases of UCLP was 3.38 (SD 1.93), for BCLP was 2.86 (SD 1.21), and for ICP was 5.75 (SD 2.06). The mean reduction in PAR score (%) in diagnoses of UCLP was −91.75 (SD 5.04), for BCLP was −93.28 (SD 3.95), and for ICP was −88.25 (SD 3.77).

**Table 1:** PAR index scores at T0 and T2 with % reduction.

			PAR index score			
Patient	Diagnosis	Surgery^a^	T0	T2	T2–T0	% reduction
1	BCLP	LF1A	41	2	−39	−95
2	ICP	LF1A, BSSO	57	6	−51	−89
3	UCLP	LF1A	47	3	−44	−94
4	BCLP	LF1A	39	5	−34	−87
5	ICP	LF1A	48	8	−40	−83
6	UCLP	LF1A	45	4	−41	−91
7	UCLP	LF1A	42	4	−38	−90
8	ICP	LF1A	39	3	−36	−92
9	ICP	LF1A	56	6	−50	−89
10	BCLP	LF1A	43	3	−40	−93
11	UCLP	LF1A	46	2	−44	−96
12	UCLP	LF1A	40	4	−36	−90
13	UCLP	LF1A, BG	54	6	−48	−89
14	UCLP	LF1A, BG	21	2	−19	−90
15	UCLP	LF1A, BG	54	4	−50	−93
16	BCLP	LF1A	38	4	−34	−89
17	UCLP	LF1A, BG	35	2	−33	−94
18	BCLP	LF1A, BG	40	2	−38	−95
19	UCLP	LF1A	40	2	−38	−95
20	BCLP	LF1A, BSSO, GP	90	2	−88	−98
21	UCLP	LF1A	41	2	−39	−95
22	UCLP	LF1A	40	2	−38	−95
23	UCLP	LF1A	41	2	−39	−95
24	UCLP	LF1A	41	2	−39	−95
25	BCLP	LF1A, BG	52	2	−50	−96
26	UCLP	LF1A	36	9	−27	−75
27	UCLP	LF1A	43	4	−39	−91
		Mean	44.8	3.6	−41.2	−91.7
		Min	21	2	−88	−98
		Max	90	9	−19	−75
		SD	11.7	2.0	11.7	4.7

^a^ Le Fort 1 advancement osteotomy (LF1A), bilateral sagittal split osteotomy (BSSO), maxillary cortico‐cancellous bone graft (BG), genioplasty (GP).

### 3.4 Cephalometric Outcomes

Mean starting ANB was −2.1° (SD 2.2) which remained relatively stable following presurgical orthodontics at T1, −2.1° (SD 2.9). Following orthognathic surgery at T2, mean ANB increased to 2.8° (SD 1.6). This can largely be attributed to the increase in mean SNA seen between T1, 77.0° (SD 6.6), and T2, 81.2° (SD 6.0) following surgical maxillary advancement. Relative incisal inclinations showed some dentoalveolar compensation of the upper incisors (UiMax) increasing from a mean of 107.5° (SD 10.1) at T0 to 114.1° (SD 6.2) at T2 which is still largely within normal reference values (Ballard 1956). Some minor lower incisor (LiMan) decompensation was achieved with a mean of 83.0° (SD 9.6) at T0, proclined to 86.3° (SD 8.1) by T2. Lower Anterior Face Height remained largely unchanged, decreasing slightly from a mean of 58% (SD 2.9) to 56.5% (SD 2.7) postsurgery. The mean starting overjet was −3.3 mm (SD 2.3) which worsened during preparation for surgery at T1 to −4.7 mm (SD 1.9). Following orthognathic surgery and postsurgical orthodontics at T2, a mean positive overjet of 2.6 mm (SD 1.3) was achieved. In only one case did a negative overjet remain at the end of treatment.

**Figure 2:**
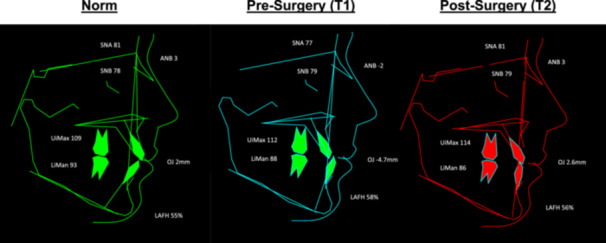
Composite value tracings of mean cephalometric values for presurgery (T1) and postsurgery (T2) compared to Caucasian reference values (Ballard 1956).

When looking at UCLP in isolation, mean T0 ANB was −1.9° (SD 2.2), increasing to 2.7° (SD 1.3) at T2. Lower incisor inclination (LiMan) was 86.5° (SD 9.8) at T0 and largely maintained at 88.2° (SD 7.9) by T2. The mean starting OJ was −3.1 mm (SD 2.0) increasing to 2.8 mm (SD 1.5) by T2. For diagnoses of BCLP, mean T0 ANB was −2.4° (SD 2.5), increasing to 3.4° (SD 1.8) at T2. Lower incisor inclination (LiMan) was 76.6° (SD 6.8) at T0 and increased slightly to 82.6° (SD 7.0) by T2. The mean starting OJ was −3.9 mm (SD 3.3) increasing to 2.9 mm (SD 1.6) by T2. Composite cephalometric tracings are demonstrated in Figure 2. Cephalometric data for all three time points is available in the supplementary data (Table S1).

**Figure 3:**
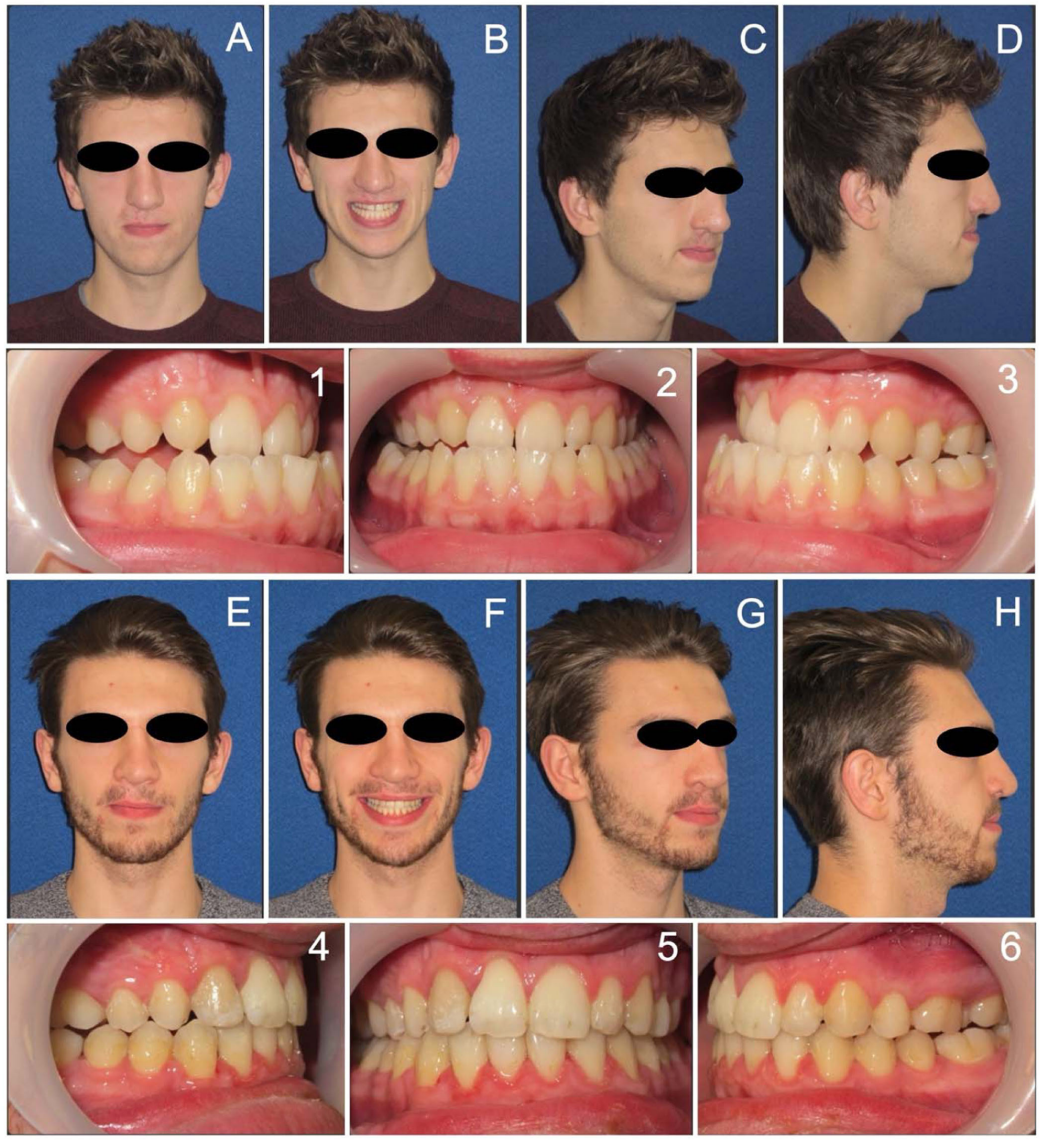
Example clinical records for an individual with a history of repaired right‐sided UCLP treated within this cohort. Extra‐ and intra‐oral photographs are shown before combined orthodontic‐orthognathic surgical treatment (Panels A–D, 1–3) and at the end of treatment, shortly after removal of orthodontic appliances (Panels E–H, 4–6). Surgery involved a Le Fort 1 osteotomy. Note the missing upper right (cleft side) lateral incisor and extraction of a premolar unit in the upper left quadrant. There is a notable improvement in midfacial and upper lip support and balancing of the Class III facial profile.

Soft tissue analysis from the lateral cephalograms measured the upper and lower lip positions relative to Ricketts Esthetic Plane (E‐line), as detailed in Table 2. The mean position of the presurgical upper lip was very retrusive at −10.4 mm compared to the Caucasian norm of −4 mm (SD ± 2.0) behind the E‐line (Ricketts 1957). Post‐orthognathic surgery, a more normalized mean upper lip position was achieved (−5.15 mm) but for some individuals, there was still a considerable lack of upper lip projection (minimum −12.5 mm). The mean position of the lower lip relative to the E‐line was within normal reference values both pre‐ (T0 = −2.13 mm) and postsurgery (T2 = −1.478). Most of the lower lip change could be attributed to soft tissue response to changes in both dental and upper lip positions postsurgery, as most individuals only had a maxillary osteotomy. An example of the occlusal and soft tissue (profile) outcome for an individual treated in this cohort is shown in Figure 3.

**Table 2:** Soft tissue assessment of upper and lower lip relative to esthetic plane.

	Upper lip (mm)			
	Min	Max	Mean	Norm for U‐lip to E‐line (mm)
Presurgical (T0)	−14.7	−6.7	−10.4	−4 (SD ± 2.0)
Postsurgical (T2)	−12.5	−0.3	−5.15	

	Lower lip (mm)			
	Min	Max	Mean	Norm for L‐lip to E‐line (mm)
Presurgical (T0)	−6.5	1.8	−2.13	−2 (SD ± 2.0)
Postsurgical (T2)	−5.1	1.6	−1.478	

One‐way ANOVA tests were used to assess the effect of primary cleft diagnosis (UCLP, BCLP, ICP) and surgical intervention (Le Fort 1 advancement only, with the addition of bilateral sagittal split osteotomy or addition of maxillary cortico‐cancellous bone grafting) on final PAR score (T2) and PAR score reduction (%). Levene's test confirmed that the assumption of homogeneity of variances was met in all instances (*p* ≥ 0.05). The ANOVA revealed a significant effect of primary cleft diagnosis on the final PAR score, *F*(2, 24) = 3.59, *p* = 0.043*, *η* ^2^ = 0.23. Post hoc comparisons using Tukey's test indicated that the mean final PAR score for BCLP cases was significantly different from ICP (*p* = 0.043, SE 1.13), but not from UCLP (*p* = 0.802, SE 0.81). Mean final PAR scores for UCLP and ICP did not differ significantly from each other (*p* = 0.66, SE 1.00). The effect of the type of surgical intervention on the final PAR score was not significant, *F*(2, 24) = 0.348, *p* = 0.709, *η* ^2^ = 0.028. The ANOVA revealed no significant effect of primary cleft diagnosis on PAR score reduction, *F*(2, 24) = 1.51, *p* = 0.241, *η* ^2^ = 0.112. The effect of surgical intervention also revealed no significant effect on mean PAR score reduction, *F*(2, 24) = 0.472, *p* = 0.63, *η* ^2^ = 0.038.

## 4 Discussion

The cleft care pathway is long, often spanning several decades, and orthognathic surgery can mark the end of active intervention, with definitive correction of facial balance. Rhinoplasty or revision speech surgery can follow as additional surgical procedures but will not change the occlusal outcome. The mean PAR score reduction of 91.6% (SD 4.7) showed great improvement in occlusion for most cases and demonstrates the standard of care that can be achieved through close interdisciplinary working. A mean pretreatment PAR score of 44.8 (SD 11.7) indicates high case complexity, comparable with other centers providing a similar cleft orthognathic service (Rolland and Deacon 2014). The significant difference in the final PAR score between BCLP and ICP cases should be interpreted with caution as the *p* value (0.043) is only just considered significant and the sample size for subgroup analyses is small and subject to individual effects. The T2 PAR score was lowest for BCLP diagnoses, with also the greatest percentage PAR score reduction. This could be attributed to both the greater starting severity of the malocclusion, and bilateral symmetry being favorably weighted in the PAR index.

There are limited reports on the occlusal outcome of cleft orthognathic surgery, but these results compare favorably with the reported PAR score outcomes of both cleft and non‐cleft populations (Rolland and Deacon 2014; Deacon et al. 2007; McMullan et al. 2003; Trimetsuntorn et al. 2020). One patient, despite having a greatly improved PAR score reduction of 75%, still had a negative overjet at the end of treatment. This was the result of challenging social circumstances and failure to attend follow‐up visits that meant postoperative settling elastics were not worn and relapse of the immediate postsurgical maxilla occurred. In the context of these individual patient factors, a decision was made to debond and accept a residual reverse overjet.

There are also essential differences to consider for patients with a cleft undergoing orthognathic surgery. The skeletal etiology is almost exclusively confined to the maxilla, with the majority of these patients having normal mandibular growth (Posnick and Ricalde 2004) or elements of mandibular retrusion (Küseler et al. 2021). This may differ from routine orthognathic surgery in non‐cleft UK populations for class III malocclusion which may more often be treated with bimaxillary surgery (Harrington, Gallagher, and Borzabadi‐Farahani 2015). Facial harmony is particularly challenging as there are additional soft tissue factors including a lack of upper lip support, deficient nasal tip projection, and paranasal hollowing. Unilateral clefting may also introduce asymmetries and there will be varying thickness of the lips, depending upon cleft type, that may necessitate further soft tissue revisions (Heliovaara et al. 2001). As has been demonstrated, surgery will most often involve a Le Fort I osteotomy with maxillary advancement (Cheung and Chua 2006). Bimaxillary surgery may be used less frequently for patients when there is a severe sagittal discrepancy or mandibular contributions to malocclusion (Cheung and Chua 2006). In some instances, segmental maxillary osteotomy may be indicated to repair residual clefts or correct transverse problems, but this was not an indication in any of the cases reported. For more severe maxillary retrusion, distraction osteogenesis has also been promoted as a technique to achieve larger advancements (Jamilian et al. 2018; Cheung and Chua 2006; Phillips, Nish, and Daskalogiannakis 2012).

Maxillary advancement appears to be a relatively stable and predictable surgical movement (Proffit, Turvey, and Phillips 2007), including in cleft populations (Parikh et al. 2021), and can improve the prominence of the upper lip, nasolabial angle, and profile convexity (Heliovaara et al. 2001). However, there are considerable variations in soft tissue response which are not comparable to non‐cleft populations. Scarring and restriction of the upper lip can lead to thinning on advancement and contribute to a class III soft tissue profile (Ewing and Ross 1993; Schendel et al. 1979). This was noted in this cohort, whereby a retrusive upper lip can persist following surgical advancement. There may also be unwanted widening of the alar base of the nose which necessitates more complex postsurgical rhinoplasty (Freihofer 1977), normally undertaken at least 6 months postoperatively.

Complete decompensation of the dental arches is not advocated presurgery as this can reverse the overjet to such an extent that larger surgical maxillary advancement or bimaxillary surgery is then required and thus a greater burden of care is imparted upon the patient. The maxillary arch is more frequently crowded and will require extractions to achieve alignment, resulting in a reduced arch length. Upper lateral incisor agenesis, particularly on the side of the cleft, will also contribute to this. Mandibular dental extractions can instead maintain the retroclination of the lower labial segment and account for the reduced maxillary arch length, preventing unopposed distal molars following surgery. Maintaining some dentoalveolar compensation will also reduce the amount of surgical advancement necessary and may limit iatrogenic effects on speech.

### 4.1 Limitations

Owing to the length of this treatment pathway and the retrospective nature of this evaluation, we did not assess the long‐term stability of occlusal and skeletal changes into the retention period. Beyond 1‐year postsurgery, the maxilla is considered to be relatively stable and so this would have normally been considered the most appropriate time to measure outcomes (Shaw et al. 2001). Postoperative records (T2) were taken at time of orthodontic debond which would have likely fallen below or near this time frame, so some consideration should be given to the scope of further surgical and orthodontic relapse that may negatively affect the long‐term occlusal outcomes reported. However, recommendations for standard outcomes in cleft care have suggested measuring outcomes at the end of active treatment (post‐orthognathic surgery with or without rhinoplasty) and this is an important time point for future study as most outcome reporting has so far been for childhood interventions (Allori et al. 2017).

As mentioned previously, there is variation in patient response by cleft type, and in particular, the soft tissues. Outcomes were measured across a composite of cleft subtypes but due to the small sample size, it was deemed unwise to carry out further subgroup analyses. It is recognized that conditions like isolated cleft palate are distinct clinical entities and not directly comparable with clefts of the lip and alveolus; however, we sought to evaluate the overall patient journey and service outcomes. Comparison with cephalometric norms were for non‐cleft Caucasian populations and is also a recognized limitation of most cephalometric studies, hence this was not a primary outcome measure for this evaluation. This study did not include a control group of patients with CL/P with Class III malocclusion who did not undergo orthodontic‐orthognathic surgery to compare occlusal outcomes. However, this would have meant denying patients' routine care. The comparison could have been made with those who declined orthognathic surgery.

Data on surgical morbidity, including infection and return to theater, are important measures of combined orthodontic‐surgical treatment. There were no incidences of patients returning to the theater or acute postoperative infection following orthognathic surgery. The most common complications of maxillary osteotomies in patients with CL/P include horizontal relapse and impairments to velopharyngeal function (Yamaguchi, Lonic, and Lo 2016). Scar tissue from primary repair and speech revision surgeries can limit mobilization and advancement of the maxilla. If the new surgical position cannot be achieved passively, soft tissue tension will contribute to postsurgical relapse. Patients with borderline velopharyngeal insufficiency (VPI) may be at greater risk of developing significant VPI following maxillary advancement surgery (Alaluusua et al. 2019; Ganoo and Sjöström 2019) but this is otherwise unpredictable (Harjunpää et al. 2019). Negative effects are evident in the early postoperative period (Pereira, Sell, and Tuomainen 2013) and appropriate assessment and review should be made within the cleft team.

### 4.2 Future Work

The final occlusal outcome of the cleft care pathway is of functional, psychosocial, and esthetic importance; however, further evidence of quality of life (QoL) improvement for these patients would be beneficial to justify this extensive treatment journey (Ganoo and Sjöström 2019). Orthognathic treatment in populations without cleft appear to show good cost‐effectiveness when outcomes are considered in the context of quality‐adjusted life years (Cunningham et al. 2003). Further work could look at cost effectiveness or utility analyses of orthognathic surgery in the context of the cleft treatment pathway. Similarly, this study could be continued with a longer follow‐up period to assess the stability of occlusal outcomes in retention; however, this will require visits and record taking outside of the normal care plan so appropriate approval and funding should be sought. Multicenter collaboration would also enable pooling of results and matching of cleft subtypes to improve sample sizes and audit outcomes (Ganoo and Sjöström 2019). Overjet, as measured with the GOSLON scale, and lateral cephalograms are the standard minimum occlusal outcome measures in cleft care (Jones et al. 2014). The PAR index weightings may not be entirely suitable for adjusted treatment goals in cleft orthodontics; however, different weightings remain to be validated for the various cleft subtypes (Jones et al. 2014) and standard weightings also enable comparison with non‐cleft populations. More patient‐centric outcome measures would also help to evidence the value of this care to both patients, their families, and the healthcare systems that support them (Tsichlaki et al. 2017). An example that could have been considered is the CLEFT‐Q (Wong Riff et al. 2017), a specifically designed research tool validated to assess patient reported outcomes of appearance, functionm and QoL for individuals affected by cleft. The CLEFT‐Q eating and drinking scale is a standard measure for patient‐reported occlusal function (Allori et al. 2017). The psychological and speech outcomes of cleft orthognathic surgery are essential in evaluating success and will be reported separately for this cohort.

## 5 Conclusions

The soft tissue and skeletal context following primary cleft repair means cleft orthognathic surgery and orthodontic treatment differs in complexity and approach to routine orthognathics. Maintaining a degree of dentoalveolar compensation during orthodontic‐surgical preparation can minimize the amount of surgical advancement required, reducing the burden of bimaxillary surgery and potential iatrogenic effects on speech. Considering these factors, these results demonstrate that cleft orthognathic treatment can still achieve occlusal outcomes comparable with non‐cleft populations. Outcome data can be used for comparison with other centers providing cleft orthognathic treatment.

## Author Contributions

Daniel Stonehouse‐Smith wrote the original draft, and analyzed and curated the data. Aida N. A. Abd Rahman was involved in data collection and methodology. Victoria Beale was involved in data collection. Haydn Bellardie conceptualized the research and methodology and provided administration and supervision of the project. All authors discussed the results and contributed to the review and editing of the final manuscript.

## Conflicts of Interest

The authors declare no conflicts of interest.

## Supporting information

Supporting information.

## Data Availability

The data that support the findings of this study are available upon reasonable request.
