# Supplementary material for: Occlusal and Cephalometric Outcomes of Cleft Orthognathic Surgery: A Retrospective Cohort Study
Source: Clin Exp Dent Res. 2024 Nov 4;10(6):e70019. doi: 10.1002/cre2.70019 (PMC11534636; doi:10.1002/cre2.70019)
Supplement: Supplementary file 1 — Supporting information. [file CRE2-10-e70019-s001.docx]

**Supplementary Data Table 3: Cephalometric values at T0, T1 and T2. ^†^Le Fort 1 advancement osteotomy (LF1A), Bilateral sagittal split osteotomy (BSSO), Maxillary cortico-cancellous bone graft (BG), Genioplasty (GP).**

|  |  |  | **T0** | | | | | | | | **T1** | | | | | | | | **T2** | | | | | | | |
| --- | --- | --- | --- | --- | --- | --- | --- | --- | --- | --- | --- | --- | --- | --- | --- | --- | --- | --- | --- | --- | --- | --- | --- | --- | --- | --- |
| **Patient** | **Diagnosis** | **Surgery†** | **SNA** | **SNB** | **ANB** | **UiMax** | **LiMan** | **IIA** | **LAFH (%)** | **OJ (mm)** | **SNA** | **SNB** | **ANB** | **UiMax** | **LiMan** | **IIA** | **LAFH (%)** | **OJ (mm)** | **SNA** | **SNB** | **ANB** | **UiMax** | **LiMan** | **IIA** | **LAFH (%)** | **OJ (mm)** |
| **1** | **BCLP** | **LF1A** | 86 | 89 | -3 | 110 | 82 | 155 | 55 | -3 | 87 | 90 | -1 | 110 | 93 | 146 | 54 | -6 | 89 | 91 | 2 | 117 | 85 | 138 | 54 | 2 |
| **2** | **ICP** | **LF1A, BSSO** | 87 | 89 | -2 | 118 | 70 | 113 | 63 | -4 | 96 | 96 | 0 | 116 | 71 | 139 | 61 | -3 | 97 | 94 | 3 | 114 | 79 | 131 | 61 | 3 |
| **3** | **UCLP** | **LF1A** | 77 | 82 | -5 | 113 | 95 | 132 | 56 | -5 | 77 | 82 | -5 | 125 | 90 | 123 | 56 | -5 | 85 | 83 | 2 | 125 | 93 | 116 | 57 | 2 |
| **4** | **BCLP** | **LF1A** | 79 | 82 | -3 | 100 | 83 | 159 | 55 | -2 | 77 | 79 | -2 | 98 | 91 | 149 | 56 | -5 | 85 | 85 | 0 | 112 | 80 | 141 | 58 | 5 |
| **5** | **ICP** | **LF1A** | 76 | 81 | -5 | 103 | 81 | 153 | 60 | -5 | 74 | 78 | -4 | 120 | 82 | 133 | 58 | -3 | 74 | 75 | -1 | 120 | 75 | 139 | 57 | 3 |
| **6** | **UCLP** | **LF1A** | 74 | 75 | -1 | 106 | 77 | 145 | 54 | -2 | 75 | 75 | 0 | 100 | 74 | 147 | 56 | -5 | 77 | 73 | 4 | 104 | 75 | 141 | 54 | 2 |
| **7** | **UCLP** | **LF1A** | 65 | 69 | -4 | 106 | 75 | 145 | 59 | -4 | 65 | 70 | -5 | 109 | 80 | 137 | 62 | -4 | 81 | 77 | 4 | 104 | 77 | 149 | 61 | 4 |
| **8** | **ICP** | **LF1A** | 79 | 79 | 0 | 103 | 88 | 148 | 57 | -3 | 76 | 76 | 0 | 109 | 98 | 131 | 55 | -4 | 78 | 74 | 4 | 106 | 97 | 135 | 53 | 2 |
| **9** | **ICP** | **LF1A** | 84 | 84 | 0 | 121 | 82 | 127 | 57 | -2 | 87 | 85 | 2 | 123 | 87 | 116 | 58 | -3 | 86 | 82 | 4 | 119 | 89 | 121 | 57 | 2 |
| **10** | **BCLP** | **LF1A** | 77 | 74 | 3 | 85 | 80 | 160 | 58 | 1 | 75 | 75 | 0 | 99 | 82 | 148 | 54 | -4 | 81 | 76 | 5 | 110 | 95 | 136 | 57 | 3 |
| **11** | **UCLP** | **LF1A** | 73 | 77 | -4 | 110 | 88 | 141 | 58 | -6 | 72 | 78 | -6 | 118 | 94 | 124 | 59 | -6 | 81 | 77 | 4 | 112 | 91 | 130 | 60 | 3 |
| **12** | **UCLP** | **LF1A** | 71 | 71 | 0 | 105 | 83 | 119 | 59 | -5 | 71 | 71 | -1 | 118 | 84 | 119 | 60 | -7 | 73 | 69 | 4 | 113 | 82 | 126 | 57 | 6 |
| **13** | **UCLP** | **LF1A, BG** | 75 | 74 | 1 | 86 | 80 | 150 | 60 | -2 | 75 | 80 | -5 | 101 | 95 | 148 | 59 | -7 | 76 | 76 | 0 | 105 | 77 | 142 | 58 | 4 |
| **14** | **UCLP** | **LF1A, BG** | 80 | 83 | -3 | 120 | 84 | 132 | 57 | 0 | 78 | 79 | -1 | 121 | 89 | 122 | 57 | -2 | 82 | 80 | 2 | 116 | 90 | 130 | 54 | 2 |
| **15** | **UCLP** | **LF1A, BG** | 87 | 85 | 2 | 105 | 97 | 152 | 57 | -2 | 85 | 85 | 0 | 116 | 93 | 122 | 57 | -2 | 88 | 84 | 4 | 118 | 87 | 132 | 55 | 2 |
| **16** | **BCLP** | **LF1A** | 78 | 80 | -2 | 96 | 68 | 160 | 65 | -4 | 79 | 81 | -2 | 112 | 75 | 134 | 65 | -5 | 72 | 69 | 4 | 112 | 73 | 139 | 55 | 5 |
| **17** | **UCLP** | **LF1A, BG** | 84 | 87 | -3 | 113 | 96 | 129 | 54 | -6 | 79 | 80 | -1 | 110 | 99 | 123 | 54 | -6 | 83 | 82 | 1 | 123 | 93 | 122 | 52 | 3 |
| **18** | **BCLP** | **LF1A, BG** | 76 | 81 | -5 | 109 | 80 | 144 | 60 | -9 | 78 | 84 | -6 | 111 | 84 | 134 | 60 | -10 | 89 | 85 | 4 | 118 | 85 | 128 | 59 | 1 |
| **19** | **UCLP** | **LF1A** | 81 | 83 | -2 | 112 | 68 | 154 | 61 | -3 | 83 | 87 | -4 | 112 | 80 | 135 | 66 | -4 | 83 | 81 | 2 | 112 | 80 | 135 | 61 | 3 |
| **20** | **BCLP** | **LF1A, BSSO, GP** | 80 | 83 | -3 | 102 | 66 | 151 | 59 | -7 | 80 | 82 | -2 | 104 | 69 | 143 | 61 | -7 | 87 | 82 | 5 | 113 | 77 | 131 | 57 | 2 |
| **21** | **UCLP** | **LF1A** | 70 | 77 | -7 | 122 | 88 | 120 | 60 | -2 | 71 | 78 | 7 | 122 | 101 | 106 | 60 | -4 | 77 | 75 | 2 | 120 | 95 | 116 | 58 | 3 |
| **22** | **UCLP** | **LF1A** | 79 | 80 | -1 | 97 | 95 | 131 | 63 | -4 | 78 | 77 | -1 | 103 | 95 | 122 | 61 | -3 | 80 | 77 | 4 | 106 | 89 | 131 | 56 | 3 |
| **23** | **UCLP** | **LF1A** | 80 | 82 | -2 | 111 | 90 | 135 | 53 | -5 | 77 | 79 | -2 | 115 | 90 | 132 | 53 | -3 | 82 | 80 | 2 | 120 | 90 | 125 | 51 | 4 |
| **24** | **UCLP** | **LF1A** | 75 | 76 | -1 | 123 | 89 | 126 | 55 | 1 | 73 | 77 | -4 | 110 | 94 | 134 | 55 | -3 | 79 | 77 | 2 | 119 | 92 | 127 | 55 | 2 |
| **25** | **BCLP** | **LF1A, BG** | 67 | 71 | -4 | 120 | 77 | 127 | 57 | -3 | 67 | 70 | -3 | 121 | 89 | 112 | 59 | -7 | 73 | 68 | 4 | 118 | 83 | 124 | 52 | 2 |
| **26** | **UCLP** | **LF1A** | 75 | 77 | -3 | 109 | 104 | 125 | 58 | -3 | 75 | 80 | -5 | 112 | 99 | 126 | 60 | -3 | 80 | 78 | 2 | 120 | 102 | 120 | 58 | 2 |
| **27** | **UCLP** | **LF1A** | 69 | 70 | -1 | 97 | 75 | 150 | 57 | -2 | 69 | 70 | -6 | 100 | 87 | 133 | 58 | -6 | 74 | 70 | 4 | 105 | 98 | 133 | 56 | -1 |
|  |  |  | **T0** | | | | | | | | **T1** | | | | | | | | **T2** | | | | | | | |
|  |  |  | **SNA** | **SNB** | **ANB** | **UiMax** | **LiMan** | **IIA** | **LAFH (%)** | **OJ (mm)** | **SNA** | **SNB** | **ANB** | **UiMax** | **LiMan** | **IIA** | **LAFH (%)** | **OJ (mm)** | **SNA** | **SNB** | **ANB** | **UiMax** | **LiMan** | **IIA** | **LAFH (%)** | **OJ (mm)** |
|  |  | **Mean** | 77.2 | 79.3 | -2.1 | 107.5 | 83 | 140.1 | 58 | -3.3 | 77 | 79.4 | -2.1 | 111.6 | 87.6 | 131 | 58.3 | -4.7 | 81.2 | 78.5 | 2.8 | 114.1 | 86.3 | 131 | 56.5 | 2.6 |
|  |  | **Min** | 65 | 69 | -7 | 85 | 66 | 113 | 53 | -9 | 65 | 70 | -6 | 98 | 69 | 106 | 53 | -10 | 72 | 68 | -1 | 104 | 73 | 116 | 51 | -1 |
|  |  | **Max** | 87 | 89 | 3 | 123 | 104 | 160 | 65 | 1 | 96 | 96 | 7 | 125 | 101 | 149 | 66 | -2 | 97 | 94 | 5 | 125 | 102 | 149 | 61 | 6 |
|  |  | **SD** | 5.8 | 5.6 | 2.2 | 10.1 | 9.6 | 13.9 | 2.9 | 2.3 | 6.6 | 6.0 | 2.9 | 8.1 | 8.8 | 11.6 | 3.3 | 1.9 | 6.0 | 6.4 | 1.6 | 6.2 | 8.1 | 8.3 | 2.7 | 1.3 |
|  |  | **Eastman Norms** | **81** | **78** | **3** | **109** | **93** | **135** | **55** |  |  |  |  |  |  |  |  |  |  |  |  |  |  |  |  |  |
|  |  | **SD** | **3** | **3** | **2** | **6** | **6** | **10** | **2** |  |  |  |  |  |  |  |  |  |  |  |  |  |  |  |  |  |
